# Competency and confidence in ECG interpretation among medical students

**DOI:** 10.5116/ijme.6372.2a55

**Published:** 2022-11-30

**Authors:** Guy Vishnevsky, Tzuriel Cohen, Yair Elitzur, Shmuel Reis

**Affiliations:** 1 Hadassah Medical School, Hebrew University, Jerusalem, Israel; 2 Department of Cardiology, Hadassah University Medical Center, Jerusalem, Israel; 3 Center for Medical Education, Hebrew University, Hadassah Faculty of Medicine, Jerusalem, Israel

**Keywords:** Electrocardiogram (ECG) interpretation, medical students, competency, confidence, Israel

## Abstract

**Objectives:**

To assess competency and confidence in ECG interpretation in medical students across years of medical school and evaluate the associations of various factors, a curriculum change, and student confidence with ECG competency.

**Methods:**

Four hundred and fourteen (414) third- to sixth-year medical students participated in this cross-sectional study conducted in 2019 in the Hebrew University of Jerusalem, Israel. A voluntary response sample of participants answered a validated, web-based questionnaire, composed of eight ECG strips. Participants were also asked about confidence and sources for ECG education and exposure. Competency and confidence across medical school years were compared using the ANOVA and chi-square tests.

**Results:**

Competency was low overall (mean score, SD (standard deviation) 3.23±1.81 out of 8), and higher in sixth-year students compared to third-, fourth- and fifth-year students (4.37±1.69 vs. 2.90±1.82, 2.90±1.54, 2.50±1.56, respectively, F_(3,337)_=24.425, p<0.0001). There was no difference between students before and after the curriculum change. Work experience in medicine was associated with competency (odds ratio (OR), 7.97; 95% confidence interval (CI), 4.03-15.77, p<0.0001). The reported confidence level was low (median 2 out of 5) and was found to be correlated with the total score achieved (r_(332)_=0.5, p<0.0001).

**Conclusions:**

Student competency was shown to be insufficient throughout medical school. Competency and confidence in ECG interpretation seem to be significantly improved by increased and repetitive exposure to ECG. Thus, strategies to facilitate better ECG skills should involve an extended focus on ECG in the undergraduate and graduate curricula and include competency-based educational programs.

## Introduction

Electrocardiography is a commonly used test for the diagnosis of diseases and electrical disorders of the heart, some of which are life-threatening conditions.^1^ The practical benefit of this test relies on the ability of the clinician to interpret the electrocardiogram (ECG) correctly. Incorrect interpretation of ECG can result in inappropriate management decisions with adverse patient outcomes.^2^ Research in medical students has reported a poor ability to accurately interpret ECG.^3–6^ Studies have demonstrated suboptimal ECG interpretive skills also among residents in training and physicians in practice.^2^

Low confidence in ECG interpretation is widely documented.^3^^-^^6^Little is known about the relationship between self-reported confidence in ECG interpretation and observed performance. A study in medical students has found confidence and competency to be correlated^7^ while another study in residents reported no correlation.^8^

In Israel, medical school graduates are expected to demonstrate ECG competency as part of their national exams.^9^ Therefore, this skill ought to be acquired and developed in the process of medical education. In the Hebrew University of Jerusalem, Israel (HUJI) faculty of medicine, ECG interpretation skills are first taught at an introductory level in a third-year physiology course. ECG interpretation is later taught in greater depth in the fourth year during the internal medicine (IM) introductory course. Students then practice ECG skills during clinical clerkships on the various wards (IM in the fourth- and sixth- years, and, optionally, a sixth-year cardiology elective with in-depth, dedicated ECG lessons). Students have written examinations involving ECG interpretation after the IM introductory course, at the end of the fourth year, and the end of the sixth year (national exams).

The traditional curriculum of the HUJI medical school (and other medical schools in Israel and the West) consists of three preclinical years of discipline-based studies coursework, followed by three years of clinical clerkships. In a recent (2016) change to the curriculum, an innovative, systems-based curriculum was implemented. This reform first focused on the preclinical studies before it affected the clinical clerkships.^10^ The new, integrated preclinical curriculum consisted of ‘blocks’ based on organ systems rather than traditional semester-long courses. Additionally, it also included an increased emphasis on physical examination, history taking, and communication skills, as well as early exposure to community medicine.

As part of the curriculum reform, in 2019, the third year of medical school was shortened. In the previous curriculum, the fourth year started with a ten-week IM introductory course; in the new curriculum, this course could be moved to the third year, allowing for more clinical exposure during medical school. Nonetheless, despite the curriculum reform, the ECG module taught during the IM introductory course has remained the same. The main difference was that students had to pass an ECG interpretation test at the end of the module.

Our primary goal was to assess whether the low competency and confidence described worldwide^3^^-^^6^are also prevalent in the HUJI medical school across years of study. We also sought to evaluate the associations of various background factors, the curriculum change, and student confidence^8^ with ECG competency.

### Methods

### Study design and participants

This study is a cross-sectional, questionnaire-based assessment of ECG interpretation skills of undergraduate medical students (n=414).  A voluntary response sample of third- to sixth-year medical students in the Hebrew University of Jerusalem, Israel participated in this study. The questionnaire was developed and validated in 2018 and data collection occurred in 2019. The questionnaire and methodology for this study were approved by the ethics committee of the Hebrew University of Jerusalem, Israel. Informed consent was obtained from all individual participants included in the study.

The third- and fourth-year cohorts represent students without any clinical experience, tested after the IM introductory course. This course, traditionally held in the fourth year of medical school, was shifted in 2019 to the third year. Therefore, fourth year students were tested before the curriculum change, while third year students were tested after the curriculum change.

### Instrument

We selected a dozen 12-lead ECGs from the Harvard Medical School sponsored ‘ECG Wave-Maven’ website,^11^ all of which were straightforward examples. Six cardiologists, including two certified electrophysiologists, were asked independently to suggest a diagnosis for each strip. ECG strips with a unanimous agreement were compared to the official interpretation and only then included in the questionnaire. During this validation process, four ECGs were excluded, and the remaining eight ECGs comprised the questionnaire.

Antiperovitch and colleagues^12^ classified ECGs into four groups using a combination of emergency/non-emergency and common/uncommon divisions. They suggested that medical students must be proficient in class A and class B ECG patterns, representing common emergency and non-emergency abnormalities. Therefore, based on this classification, we included four strips presenting common emergency cardiovascular conditions (acute ST-elevation myocardial infarction (STEMI), ventricular tachycardia (VT), supraventricular tachycardia (SVT) and a markedly prolonged QT (QTc=600 milliseconds)) and three common non-emergency ECG abnormalities (atrial fibrillation (AF), left bundle branch block (LBBB) and pacemaker rhythm). We also included a normal ECG.

The questionnaire began with five open-ended questions regarding the primary sources of the participants for education in ECG interpretation. Participants were asked whether they acquired their ECG interpretive skills through the following sources: attendance in ECG classes, teaching during clinical clerkships, and self-study using printed materials or web-based sources. They were also asked about previous work experience in medicine, such as a physician’s assistant (a position often filled by medical students in Israel) or a paramedic (EMS or military). Participants then had to specify their year of study. The eight ECGs followed this part.

A brief clinical scenario was provided for each ECG, which consisted solely of age, gender, and chief complaint.^13^ For each ECG, study participants were instructed to determine whether it showed normal findings, write a primary diagnosis in case the ECG was abnormal and indicate their degree of confidence in their diagnosis using a Likert scale from 1 to 5 (where 1 represented a complete guess and 5 represented absolute confidence). Localization of the abnormality was required for the first ECG, featuring an acute anterior wall STEMI, and for it only. The questionnaire concluded with a question about the overall confidence level on a five-point Likert scale. There was no time limit.

Each answer was graded 0 / 0.5 / 1 points (0 = incorrect, 0.5 = partially correct, 1 = correct) using a prespecified answer key derived from the validation process described above. Thus, the maximum total score achievable was 8, and the passing score was considered 5, which is consistent with competency greater than 60% - a commonly used threshold for a passing grade on exams.

### Data collection

Participants were gathered in a lecture hall. They received a link to a web-based version of the questionnaire and answered it. All of the participants were supervised during the entirety of the questionnaire. To allow comparisons across groups, we used identical questionnaires in all groups, but each group completed them on different dates, spaced months apart. To avoid contamination between groups, we created a unique link for each group, blocked access to the questionnaire between the different dates, and did not provide any feedback or answers.

### Data analysis

Reliability, or internal validity, was measured with Cronbach’s Alpha. One way ANOVA with a Scheffe correction for post-hoc analyses was used to compare scores between years of study. The chi-square test was used to compare pass rates, and to investigate the associations between scores and year of study for each ECG strip. A Bonferroni correction was used for multiple pairwise comparisons.

A chi-square univariate analysis identified variables that were significantly associated with a passing score, which were subsequently entered in the multiple regression model. The multiple logistic regression model was used to assess which reported sources of knowledge were significantly associated with competency, where the response variable was a passing score in the questionnaire. Spearman's rho correlation test was used to explore the correlation between the reported overall confidence level and the total score in the questionnaire.

Statistical analysis was performed with IBM SPSS Statistics 25, and statistical significance was defined as p<0.05.

## Results

A total of 414 medical students consented to participate in this study, of which 341 students completed the questionnaire. The Cronbach’s Alpha for the eight-item questionnaire was 0.671. Most students stated to have acquired skills in ECG interpretation from attending regular ECG classes (n=293, 71%) or formal ECG teaching during clinical clerkships (n=150, 67% of fifth- or sixth- year student, excluding preclinical years). Reported clerkships were mainly in IM and cardiology (n=48, 32% and n=45, 30%, respectively). More than half of students (n=222, 54%) reported self-learning as a significant source of knowledge for ECG interpretation, with printed materials, such as textbooks, being reported more often (n=164, 40%) than online resources (n=98, 24%). Some students (n=75, 18%) reported that they gained their knowledge from work experience in medicine. Of these students, most (n=44, 59%) were physician assistants, and a few (n=6, 8%) were paramedics.

Scores across the study population were found to be low - overall mean, SD of 3.23±1.81 (Table 1). The mean score for sixth-year students was 4.37±1.69, which was significantly higher than the mean scores for third-, fourth-, and fifth-year students (4.37±1.69 vs. 2.90±1.82, 2.90±1.54, 2.50±1.56, respectively, F_(3,337)_=24.425, p<0.0001 for all; Table 1). Similarly, sixth-year students had a significantly higher pass rate than the other groups (45% vs. 18%, 11%, and 15%, respectively χ^2^(3, N=341) =38.876, p<0.0001; Table 1). There were no other statistically significant differences between the years of study regarding overall competency, including in a direct comparison between third- and fourth-year students (comprising the before and after curriculum change groups).

Most pathological ECGs (Table 2, n=2062, 92%) were classified as such; students were less successful in identifying the normal ECG strip (n=221, 65%). There were no statistical differences between years of study in the overall ability to distinguish normal from abnormal ECGs. Nevertheless, when ECGs were classified correctly as abnormal, sixth-year students had a significantly higher rate of correct diagnoses than third-, fourth-, and fifth-year students (54% vs. 46%, 42% and 40%, respectively χ^2^(3, N=2189)=31.481, p<0.0001; Table 2). Compared to the other groups, sixth-year students had a significantly higher percentage of correct answers on 6 of the 8 ECGs (Table 2).

**Table 1:** Competency and confidence according to years of study

Year of study	n	**C**ompleted questionnaire (%)	Passed (%)	Mean (95% CI), SD	Median^*^
3	88	63 (71.6)	11 (17.5)	2.90 (2.45–3.36), 1.82	2
4	103	83 (80.6)	9 (10.8)	2.90 (2.56–3.23), 1.54	2
5	115	93 (80.9)	14 (15.1)	2.50 (2.18–2.82), 1.56	2
6	108	102 (92.4)	46 (45.1)	4.37 (4.04–4.70), 1.69	3
Total	414	341 (82.4)	80 (23.5)	3.23 (3.04–3.42), 1.81	2

CI: Confidence Interval; *Median of reported overall confidence

As shown in Tables 1 and 2, notwithstanding the curriculum change, third- and fourth-year students had no significant differences in all seven abnormal ECGs scores, total scores, and pass rates.

In each group, the highest competency was observed in the STEMI ECG strip (total competency of 81%, n=310; Table 2). The most misread abnormalities were long QT, LBBB, SVT and pacemaker rhythm (total competency of 10%, 29%, 30%, and 31%, respectively; Table 2).

**Table 2:** Correct answers for each ECG according to years of study

Diagnosis		Year of Study				Total n (%)	p-value^*^
		3 n (%)	4 n (%)	5 n (%)	6 n (%)		
Any abnormal ECG identification		385 (96)	482 (91)	560 (91)	635 (93)	2062 (92)	0.743
Common Emergencies	STEMI	54 (71)	60 (67)	93 (84)	103 (97)	310 (81)	< 0.0001
	VT	39 (59)	34 (41)	43 (42)	76 (74)	192 (54)	< 0.0001
	SVT	15 (24)	22 (27)	23 (24)	45 (44)	105 (30)	< 0.0001
	Long QT	2 (3)	4 (5)	3 (3)	26 (26)	35 (10)	< 0.0001
	Total	110 (41)	120 (36)	162 (39)	250 (60)	642 (45)	< 0.0001
Common Abnormalities	LBBB	14 (22)	25 (30)	15 (14)	48 (47)	102 (29)	< 0.0001
	Atrial fibrillation	35 (60)	38 (46)	36 (41)	54 (55)	163 (50)	0.263
	Pacemaker	17 (29)	18 (22)	10 (11)	59 (58)	104 (31)	< 0.0001
	Total	66 (37)	81 (33)	61 (22)	161 (53)	369 (36)	< 0.0001
Correct abnormal ECG diagnosis		176 (46)	201 (42)	223 (40)	411 (54)	1011 (46)	< 0.0001
Normal ECG diagnosis		29 (49)	64 (80)	57 (59)	71 (69)	221 (65)	0.704

* p-value calculated for years 3, 4 and 5 vs. year 6.

**Table 3:** Association between sources of ECG knowledge and competency in ECG interpretation

Factor	Univariate logistic regression		Multiple logistic regression	
	OR (95% CI)	p-value	OR (95% CI)	p-value
Attendance in regular ECG classes	0.51 (0.30–0.85)	0.010	1.07 (0.57–1.99)	0.838
ECG teaching during clinical clerkships	2.75 (1.65–4.60)	<0.0001	1.36 (0.62–2.97)	0.444
Self-learning	0.78 (0.47–1.28)	0.320		
Self-learning, printed materials	1.04 (0.63–1.74)	0.871		
Self-learning, web-based sources	0.64 (0.34–1.20)	0.163		
Work experience in medicine	7.78 (4.28–14.14)	<0.0001	7.97 (4.03–15.77)	<0.0001

OR: Odds Ratio; CI: Confidence Interval.

Univariate regression analysis showed (Table 3) that except self-learning methods, all other sources of knowledge were associated with competency in ECG interpretation, including attendance in regular classes (χ^2^(1, N=341)=6.675, OR=0.51 [95% CI 0.30-0.85], p=0.01), teaching during clinical clerkships (χ^2^(1, N=341)=15.555, OR 2.75 [95% CI 1.65-4.60], p<0.0001) and work experience in medicine (χ^2^(1, N=341)=53.534, OR=7.78 [95% CI 4.28-14.14], p<0.0001). However, a multiple logistic regression model showed that, holding all other variables constant, previously having work experience in a medical position was the only factor associated with a passing score (OR=7.97 [95% CI 4.03-15.77], p<0.0001; Table 3). Subgroups of work experience (mostly physician assistants and paramedics) were too small to compare.

Students tended to report low confidence in their diagnoses. On five ECGs, the median reported level of confidence was 3 (Normal, STEMI, VT, SVT, LBBB), and on three ECGs, the median was 2 (Long QT, AF, pacemaker). The ECG presenting STEMI was the strip with the highest percentage of participants reported complete confidence about their diagnosis (14%). For sixth-year students, the median confidence was 3, and for the rest of the students, the median was 2 (Table 1). A statistically significant correlation was found between the reported overall confidence level and the total score achieved (r_(332)_ = 0.5, p<0.0001; not shown).

## Discussion

In this study, we found that Israeli medical students in HUJI have low competency in ECG interpretation. Performance scores were very low throughout, even for those on the verge of completing their course of study. Since the ECGs in the questionnaire were specifically chosen as examples of conditions that medical students should be able to interpret^12^ - all of which are either quite common or life-threatening conditions - higher scores would be expected. Participants were usually able to recognize the abnormality of an ECG but often failed to specify the correct disorders.

The questionnaire was demonstrated to be reliable and had an acceptable Cronbach’s Alpha (α=0.671).^14^^,^^15^ We find this value to be reassuring in light of the literature.^16^^,^^17^ Moreover, the questionnaire included relatively few items, which likely caused an underestimation and negatively affected the Cronbach’s Alpha.^14^^,^^18^

The finding of poor performance in ECG interpretation among medical students is consistent with previous studies. Research in final-year students found ECG interpretation accuracy rates of 54% among 46 Irish students,^5^ 52% among 52 New Zealand students,^4^ 26% among 74 South Asian students,^19^ 37% among 168 American students,^7^ and 60% among 156 Saudi Arabian students.^6^ However, the comparison of different studies concerning ECG interpretation is difficult since ECGs used for testing and methodologies vary. It should be noted that there are neither worldwide nor national guidelines defining satisfactory ECG competency.

We hypothesized that students of each year of study would present greater competency compared to students of a lower year of study, as clinical skills are developed and accumulated during the process of medical education.^7^^,^^20^ Indeed, sixth-year students showed a significantly improved ability to interpret ECG, but there were no other statistically significant differences between the years of study. This contradicts findings by Kopeć and colleagues, who compared students at different stages of their clinical studies and reported that competency in ECG interpretation did not improve between fourth- to sixth-year students.^3^ The inconsistent results might be explained by differences in teaching and assessment methods, which can impact performance significantly.^12^^,^^21^

It should be noted that currently, the fifth year does not include an IM clerkship, or another clerkship with significant ECG exposure. This may explain the similarities in competency between fourth- and fifth-year students. The better performance of sixth-year students is probably due to accumulated experience, ECG teaching and clinical exposure during sixth-year clerkships in addition to self-learning in preparation to the national exam at the end of this year. This supports the current clinical studies curriculum. It should be also noted that physician assistants are usually fifth- or sixth- year students.

Third- and fourth-year students were at the same stage of medical training because of the newly implemented curriculum change. In the context of ECG training, both groups were taught the same module, except third-year students had to pass an ECG interpretation test at the end of the module. In medical education, testing has been shown to promote learning and improve long-term knowledge retention.^22^ Nevertheless, there were no differences in the observed competency of students before and after the curriculum reform. Except for recommendations to use tests for the improvement of ECG interpretation skills,^12^ studies that clarify the role of test-enhanced learning on ECG interpretation specifically remain scarce.

We therefore could not draw any conclusions regarding the effect of the curriculum change on ECG competency. Students tested before and after the change were tested at an early stage, before the possible benefits of the new curriculum (chiefly, longer clinical exposure) took place. The impact of the extended clinical exposure on ECG interpretation skills might be better appreciated at the completion of medical school.

We have found that some pathologies were more likely to be misdiagnosed. It could prove vital to identify these gaps in ECG competency and address them. As the level of competency in ECG interpretation was previously found to be correlated with the degree of ECG exposure^7^^,^^23^ it is expected that low competency will be observed in ECG patterns that are less encountered by students, and vice versa. For example, the highest competency was observed in the STEMI ECG - a frequently encountered and taught abnormality. We only used ECG abnormalities classified as common^12^ that students would have reasonably seen before. The higher competency among sixth-year students in almost all strips further supports the merits of repeated exposure, such as spiral learning and other repetition-based teaching strategies.

We have shown that occupational experience in medicine significantly improved competency in ECG interpretation, regardless of the type of job. It is known that the amount of practice time and the number of ECG cases practiced are correlated with diagnostic accuracy.^23^

Both self-learning and formal teaching failed to improve ECG interpretation skills. Studies in this area have shown contradictory results.^24^ While Kopeć and colleagues^3^ reported that self-learning was an important determinant of ECG interpretation and regular ECG classes did not influence competency, a prospective randomized study^25^ found that self-learning was less effective than formal teaching. Our results are consistent with the idea of improvement through spiral learning, which is more prevalent in clinical, hands-on settings rather than a lecture-based module and self-learning.

In our study, medical students appeared to have low confidence in their ability to interpret ECG. This observation is consistent with previous studies in medical students.^4^^,^^5^^,^^7^ We have found a significant positive correlation between self-reported confidence regarding ECG interpretation and the total score achieved in the questionnaire. A previously published study in medical students has shown similar results, with the degree of confidence associated with the mean number of ECGs answered correctly.^7^

It is unclear how confidence can be fostered as an integral part of medical education. As the level of confidence in the performance of a specific skill is correlated with the clinical experience in this area,^26^ it seems that the addition of more opportunities to practice ECG cases to the curriculum may improve confidence. We found relatively higher levels of confidence among the sixth-year students, once again highlighting the importance of repetition and of accumulated experience.

We find the extent of ECG exposure to be a significant factor in the acquisition of interpretation skills. It may be necessary to incorporate more ECG practice opportunities into both undergraduate and graduate curricula (particularly in the fifth year, that lacks any ECG-related content).

Our study has several strengths. We included participants from different years of medical school, enabling us to examine the progression of ECG interpretation competency through the process of medical education. Our sample size was relatively large, strengthening our conclusions. Our questionnaire included validated, diverse, and relevant ECGs and clinical scenarios. Notably, it is the first study (from our institution and otherwise) comparing students before and after the described integrative curriculum reform. Finally, we have identified a manageable factor that we believe had a significant effect on the confidence and competency of the participants in our study.

There are also several limitations to this study. As with all studies based on questionnaires, a measurement bias might affect the results due to known and unknown flaws of the questionnaire. For example, the choice of our ECG strips and the grading system are subject to bias.

Since the study included only the results of one institution, it does not necessarily represent national sampling. Hence, the extent to which the findings of this study can be generalized may be limited. As participation in our study was voluntary, our sample may be biased and not represent all HUJI medical students. (For example, it is possible that students who were more competent at ECG interpretation were more likely to choose to participate in the study.) If such bias does exist, our findings are even more concerning considering the poor competency that was observed.

The concept of regression toward the mean should be considered as well, as the performance of medical students in our study may seem extremely low. However, this finding is relatively consistent through the different years of study. National exam results are similar between all medical schools in Israel. Most importantly, low ECG competencies have been widely seen in studies from other countries.

Another previously discussed limitation is that 3rd and 4th year students were before their clinical studies, limiting our ability to investigate the effect of the extended clinical exposure that resulted from the curriculum change on their ECG interpretation skills.

## Conclusions

Our study suggests that medical students do not appear to be prepared to interpret ECG abnormalities, including life-threatening conditions, even at the completion of their clinical training. The ECG teaching was insufficient, which is likely to be also true for other institutions. This inadequacy, if not remedied, may result in incorrect medical management decisions and adverse patient outcomes.

Competency and confidence in ECG interpretation seem to be significantly improved by increased and repetitive exposure to ECG, in occupational, instructional and other settings. Thus, strategies to facilitate better ECG skills should involve an extended focus on ECG in the undergraduate and graduate curricula and include competency-based educational programs.

### Conflict of Interest

The authors declare that they have no conflict of interest.
